# Dietary supplementation of arachidonic acid increases arachidonic acid and lipoxin A_4_ contents in colon, but does not affect severity or prostaglandin E_2_ content in murine colitis model

**DOI:** 10.1186/1476-511X-13-30

**Published:** 2014-02-10

**Authors:** Norifumi Tateishi, Saki Kakutani, Hiroshi Kawashima, Hiroshi Shibata, Ikuo Morita

**Affiliations:** 1 Institute for Health Care Science, Suntory Wellness Ltd, 1-1-1 Wakayamadai, Shimamoto, Osaka 6188503, Japan; 2 Department of Cellular Physiological Chemistry, Graduate School, Tokyo Medical and Dental University, Tokyo, Japan

**Keywords:** Arachidonic acid, Colitis, Prostaglandin E_2_, Lipoxin A_4_

## Abstract

**Background:**

Arachidonic acid (ARA) is an essential fatty acid and a major constituent of biomembranes. It is converted into various lipid mediators, such as prostaglandin E_2_ (PGE_2_) and lipoxin A_4_ (LXA_4_). The effects of dietary ARA on colon maintenance are unclear because PGE_2_ has both mucosal protective and proinflammatory effects, and LXA_4_ has an anti-inflammatory role. Our objective is to clarify the effects of dietary ARA on an experimental murine colitis model.

**Methods:**

C57BL/6 mice were fed three types of ARA diet (0.075%, 0.15% or 0.305% ARA in diet), DHA diet (0.315% DHA) or control diet for 6 weeks, and were then administered dextran sodium sulphate (DSS) for 7 days to induce colitis. We evaluated colitis severity, fatty acid and lipid mediator contents in colonic tissue, and the expression of genes related to lipid mediator formation.

**Results:**

ARA composition of colon phospholipids was significantly elevated in an ARA dose-dependent manner. ARA, as well as DHA, did not affect colitis severity (body weight loss, colon shortening, diarrhea and hemoccult phenomena) and histological features. PGE_2_ contents in the colon were unchanged by dietary ARA, while LXA_4_ contents increased in an ARA dose-dependent manner. Gene expression of cyclooxygenase (COX)-1 and COX-2 was unchanged, while that of 12/15-lipoxgenase (LOX) was significantly increased by dietary ARA. ARA composition did not correlate with neither colon length nor PGE_2_ contents, but significantly correlated with LXA_4_ content.

**Conclusion:**

These results suggest that dietary ARA increases ARA and LXA_4_ contents in colon, but that it has no effect on severity and PGE_2_ content in a DSS-induced murine colitis model.

## Background

Polyunsaturated fatty acids (PUFAs), such as arachidonic acid (ARA) and docosahexaenoic acid (DHA), are natural nutrients present in common foodstuffs (e.g., egg yolk, meat and fish oil). ARA is an n-6 essential fatty acid and is a major constituent of biomembranes. One of its physiological roles is acting as the starting substrate for various lipid mediators, such as prostaglandins (PGs), lipoxins (LXs), leukotrienes, endocannabinoids and epoxyeicosatetranoates [1-3].

Colitis is a major inflammatory disease in digestive organs [4]. Administration of non-steroidal anti-inflammatory drugs (NSAIDs) is avoided in colitis patients because they inhibit cyclooxygenase and reduce the production of PGE_2_, which has an essential role in the protection of gastrointestinal mucosa [5-7]. Furthermore, lipoxin A_4_ (LXA_4_), synthesized from ARA by 12/15-lipooxgenase and 5-lipoxygenase, has been identified to have anti-inflammatory potential and to play a role in the “resolution phase” that follows and terminates the “inflammation phase” [2,8]. On the other hand, PGE_2_ is known to be proinflammatory for various acute inflammations [1]. Therefore, the effects of ARA and the ARA-derived lipid mediators on colitis are complicated.

It has been clarified that dietary ARA affects the ARA composition of phospholipids in humans and animals [9-13]. We recently reported that ARA supplementation increases plasma ARA but does not increase ARA-derived lipid mediators or clinical parameters, including inflammation parameters such as C-reactive proteins, interleukin-6 (IL-6) and tumor necrosis factor-α (TNF-α), in healthy Japanese elderly individuals [12]. However, the effects of dietary ARA in pathological conditions are not fully understood. In the case of colitis, Ramakers et al. reported that intake of an ARA-enriched diet did not affect colonic inflammation, as compared with intake of fish oil- or oleic acid-enriched diets in mice with experimental colitis [14]. However, changes in the contents of ARA-derived lipid mediators in the colitis model have not been investigated. Therefore, in order to clarify the effects of dietary ARA on a murine colitis model, we evaluated the effects of ARA at various doses on colitis severity, determined the contents of ARA and ARA-derived lipid mediators, and assessed the expression of genes related to these lipid mediators.

## Materials and methods

### Animals, diets and experimental design

Experiments were approved by the Animal Care and Use Committee of Suntory Holdings, Ltd. (Osaka, Japan), and we followed the Guidelines for Animal Care and Use of Suntory Holdings, Ltd. Sixty 3-week-old female C57BL/6 mice were obtained from Charles River Japan (Yokohama, Japan). Mice were housed under standard conditions and had free access to water and diet.

We used five types of diet based on AIN-76 rodent diet (Table 1). The composition of the diets was the same as that of AIN-76, except for lipids. The lipids used in the present study were ARA-enriched triacylglycerol (SUNTGA40S) (lot no. 100120A1; Nippon Suisan Kaisha Ltd., Tokyo, Japan), fish oil (DHA-27 W) (lot no. 1003251; Maruha Nichiro Food Corporation Ltd., Tokyo, Japan), palm oil, soybean oil, and linseed oil (Showa Cousan Ltd., Osaka, Japan). ARA composition was approximately 42.5% in ARA-enriched triacylglycerol, which was used in diets for the ARA(L), ARA(M) and ARA(H) groups. DHA and eicosapentaenoic acid (EPA) compositions were approximately 26.8% and 6.8%, respectively, in fish oil, which was used in a diet for the DHA groups. Palm oil, soybean oil and linseed oil contained no ARA, DHA or EPA, and were used to adjust the amounts of total lipids, total saturated fatty acids (S), total monounsaturated fatty acids (M), total polyunsaturated fatty acids (P), total n-6 fatty acids and total n-3 fatty acids to similar levels in the five diets, respectively (Table 1). Experimental diets were stored at 4°C and protected from light to prevent oxidation.

**Table 1:** Fatty acid composition of the diets

**Fatty acids**	**CON**	**ARA(L)**	**ARA(M)**	**ARA(H)**	**DHA**
	**g/100 g fatty acids**				
16:0 palmitic acid	27.6	28.1	28.2	28.3	28.3
18:0 stearic acid	4.2	4.3	4.6	4.9	4.4
18:1(n-9) oleic acid	32.1	31.5	31.0	29.6	30.9
18:2(n-6) linoleic acid	23.5	21.1	19.3	15.6	21.4
18:3(n-3) α-linolenic acid	11.3	11.4	11.2	11.3	2.3
20:3(n-6) dihomo-γ-linolenic acid	0.0	0.1	0.3	0.5	0.0
20:4(n-6) arachidonic acid	0.0	1.5	3.0	6.1	0.5
20:5(n-3) eicosapentaenoic acid	0.0	0.0	0.0	0.0	1.6
22:6(n-3) docosahexaenoic acid	0.0	0.0	0.0	0.0	6.3
Others	1.3	2.0	2.4	3.7	4.3
Total	100	100	100	100	100
PUFA	35.1	34.6	34.5	34.6	33.4
MUFA	32.9	32.5	31.8	30.4	33.6
SFA	32.0	33.0	33.8	35.0	33.0
n-6/n-3	2.1	2.0	2.1	2.0	2.2

Fatty acid composition of the experimental diets. Numbers in the table are expressed in percent except for the n-6/n-3 ratio. PUFA: polyunsaturated fatty acid; MUFA: monounsaturated fatty acid; SFA: saturated fatty acid. Note that each diet comprised equal amounts of each fatty acid and had the same n-6/n-3 ratio.

**Figure 1:**
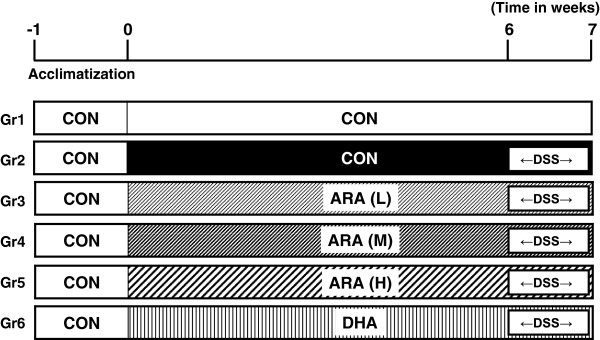
Experimental procedure for DSS-induced colitis model in the present study.

After a 1-week acclimation period, mice were randomly assigned to six groups of 10 mice, and received one of the five diets described above for 6 weeks before colitis induction (Figure 1). Body weight was recorded every third or fourth day during the first 6 weeks. Colitis was then induced by administration of 2.0% (w/v) dextran sulfate sodium (DSS, average molecular weight 36–50 kDa) (MP Biomedical, Santa Ana, CA) in drinking water for 7 days. After colitis induction, body weight, stool consistency and occult blood in the stool were monitored daily [15,16]. Diarrhea was scored as follows: 1, normal; 2, loose stools; 3, soft mud-like stools; and 4, watery diarrhea. Hemoccult was scored as follows: 1, normal; 2, trace positive; 3, strong positive; and 4, gross bleeding. After the 7-day DSS treatment, all mice were anesthetized with diethyl ether and killed via cardiac puncture and exsanguination. Hematocrit (HCT) and white blood cell (WBC) number were determined using a glass capillary and blood cell counter. Plasma was obtained by centrifugation at 8000 rpm for 10 min at 4°C and stored at -80°C. Total length and weight of colon were measured as an indicator of the severity of inflammatory bowel disease. The colon was divided into the proximal, middle and distal positions. The first and second positions were frozen in liquid nitrogen and used for analyses of fatty acid, lipid mediator and gene expression. The third position of these were fixed in 10% neutral buffered formalin and used for histological evaluation.

### Histological analysis

The distal position of the colon fixed in 10% neutral buffered formalin were processed for histology and stained with hematoxylin and eosin. Histological scoring for inflammation and crypt destruction was performed in a blinded manner by a pathologist, as described previously [15].

### Fatty acid analysis of the diets and colon homogenates

Lipids in the diets and colon were extracted and purified by the method of Folch et al. [17]. Lipids in colon were separated into phospholipids (PL) and other lipid fractions by thin-layer chromatography using silica gel 60 (Merck, Darmstadt, Germany). The solvent system consisted of hexane/diethyl ether (7/3, v/v). Fatty acid residues in extracted lipids or separated phospholipids were analyzed by the method of Sakuradani et al. [18]. Briefly, each lipid fraction was incubated with an internal standard (pentadecanoic acid) in methanolic HCl at 50°C for 3 h to transmethylate fatty acid residues to fatty acid methyl esters, which were extracted with *n*-hexane and analyzed by capillary gas–liquid chromatography.

### Analysis of lipid mediators of colon homogenates

Prostaglandin E_2_ (PGE_2_), PGE_2_-d4, lipoxin A_4_ (LXA_4_), LXA_4_-d5, leukotriene B_4_ (LTB_4_) and LTB_4_-d4 were obtained from Cayman Chemical (Ann Arbor, MI, USA). The methods for extraction and analysis of lipid mediators were as reported previously [19], with some modification. Briefly, colon tissue frozen in liquid nitrogen was ground using a Multi-Beads Shocker MB701(S) (Yasui Kikai, Osaka, Japan), and homogenized with ice-cold ethanol. A fixed amount of PGE_2_-d4, LXA_4_-d5 and LTB_4_-d4 was added to all homogenates as an internal standard. After centrifugation, each supernatant was dried by centrifugal evaporation, and residues were dissolved in methanol, washed and concentrated with SPE cartridges (Empore disk cartridge C18 SD; 3 M, St. Paul, MN), as described previously [19]. An Agilent 1200 HPLC system (Agilent Technologies, Santa Clara, CA) equipped with a Cadenza CD-C18 column (3 mm, 2 mm i.d. × 150 mm; Imtakt, Kyoto, Japan) and quadruple linear ion trap hybrid mass spectrometer, 4000 Q TRAP, with electrospray interface (Applied Biosystems/MDS SCIEX, Concord, Canada) was used for quantification. The mass spectrometer was operated in negative ion mode with selected reaction monitoring. PGE_2_ and PGE_2_-d4 were detected by monitoring mass transitions at *m/z* 351 → 271 for PGE_2_ and *m/z* 355 → 275 for PGE_2_-d4 at a collision energy of -24 V. The quantitative range of PGE_2_ was 0.3 – 100 ng/injection. LXA_4_ and LXA_4_-d5 were detected by monitoring mass transitions at *m/z* 351 → 115 for LXA_4_ and *m/z* 356 → 115 for LXA_4_-d5 at a collision energy of -22 V. The quantitative range of LXA_4_ was 3 – 1000 pg/injection. LTB_4_ and LTB_4_-d4 were detected by monitoring mass transitions at *m/z* 335 → 195 for LTB_4_ and *m/z* 339 → 197 for LTB_4_-d4 at a collision energy of -24 V. The quantitative range of LTB_4_ was 0.6 – 200 pg/injection.

### Quantitative real-time polymerase chain reaction (QRT-PCR)

In order to assess the effects of DSS treatment and diet on gene expression, we selected DSS-untreated, DSS-treated CON, ARA(H) and DHA diet groups. For mRNA analysis, total RNA from colonic tissues stored at -80°C was extracted using Isogen (Nippon Gene Co., Ltd., Toyama, Japan) and purified with an RNeasy mini kit (Qiagen GmbH, Hilden, Germany). Total RNA (2.0 *μ*g) was reverse-transcribed with random primers using High-Capacity cDNA Reverse Transcription Kits (Applied Biosystems, Foster City, CA), in accordance with the recommendations of the manufacturer. To quantify gene expression, cDNA was amplified for various gene targets by QRT-PCR using the ABI PRISM 7900 Sequence Detection System (Applied Biosystems). All primers and probes used were purchased as TaqMan Gene Expression Assays: cytosolic phospholipase A2 (cPLA2, Mm00447040_m1), cyclooxygenase-1 (COX-1, Mm00477214_m1), cyclooxygenase-2 (COX-2, Mm00478374_m1), arachidonate 5-lipoxygenase (5-LOX, Mm01182747_m1), and arachidonate 12/15-lipoxygenase (12/15-LOX, Mm00507789_m1) (Applied Biosystems). PCR results were analyzed with ABI SDS software (Applied Biosystems). Relative expression levels of the genes in each sample were determined by the Comparative Ct Method. Expression assays for each gene were normalized against glyceraldehyde-3-phosphate dehydrogenase (GAPDH, Mm99999915_g1) and expressed as fold change relative to that of the DSS-untreated group.

### Statistical analysis

Data are presented as means ± SD. Data were analyzed by one-way ANOVA followed by Dunnett or Steel multiple comparisons. Correlation analyses were performed using the Spearman correlation test. Values of P < 0.05 were considered to be statistically significant.

## Results

### Fatty acid composition of the colon homogenates

**Figure 2:**
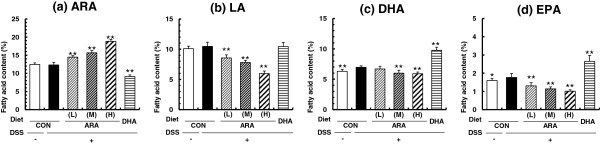
**Fatty acid composition of phospholipids ((a) ARA, (b) LA, (c) DHA and (d) EPA) in the colon from dextran sodium sulphate colitis mice fed a CON, ARA(L), ARA(M), ARA(H) and DHA diet.** Data are represented as means ± SD. *p < 0.05, **p < 0.01 versus DSS-treated CON diet group (n = 10).

Figure 2 shows the fatty acid composition of the colon phospholipids. ARA and LA composition was unchanged by the DSS treatment whereas DHA and EPA composition was increased by the DSS treatment. Dietary ARA significantly increased the ARA composition of the colon phospholipids in a dose-dependent manner. The ARA composition in the CON and ARA(H) groups was 12.3 ± 0.7% and 18.8 ± 0.6%, respectively. Dietary ARA inversely decreased DHA and LA composition in an ARA dose-dependent manner. In the DHA group, DHA composition was high (9.8 ± 0.5%) and ARA composition was low (9.1 ± 0.5%), as compared to the CON group (DHA composition, 6.7 ± 0.3%; and ARA composition, 12.3 ± 0.7%).

### Effects of dietary ARA on DSS-induced colitis

**Figure 3:**
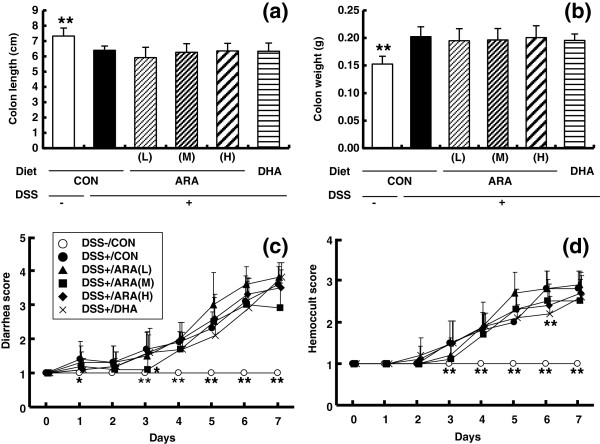
**Induction of colitis treated with 2% DSS and the effects of CON, ARA(L), ARA(M), ARA(H) and DHA diet on colitis disease severity.** Mice consumed each diet for 42 days before DSS colitis induction. Colitis was induced by 2% (w/v) DSS in drinking water for 7 d. Disease severity was assessed by colon length **(a)**, colon weight **(b)**, diarrhea score **(c)** and hemoccult score **(d)**. Data are represented as means ± SD. *p < 0.05, **p < 0.01 versus DSS-treated colitis group (n = 10).

Before colitis induction with DSS, mice were fed CON, ARA(L), ARA(M), ARA(H) or DHA diet for 6 weeks. Body weight in the ARA(M), ARA(H) and DHA groups was significantly lower than in the CON group during the preceding period (Additional file 1: Figure S1). Mice exposed to 2% DSS for 7 days showed typical symptoms of acute colitis, such as a decrease in colon length (DSS-untreated CON 7.5 ± 0.5 cm, DSS-treated CON 6.4 ± 0.3 cm) (Figure 3a) and an increase in colon weight (0.15 ± 0.01 g versus 0.20 ± 0.02 g) (Figure 3b). Severe diarrhea (Figure 3c), hemoccult (Figure 3d) and a decrease in body weight (Additional file 2: Figure S2 and Additional file 3: Table S1) were also observed, with HCT values decreasing and spleen weight increasing significantly (Additional file 3: Table S1). Dietary ARA did not affect any colitis symptoms (Figure 3a-d, Additional file 3: Table S1). DHA administration improved hemoccult score at day 6 after DSS induction (Figure 3d) and spleen weight (Additional file 3: Table S1), but had no effects on the other parameters.

**Figure 4:**
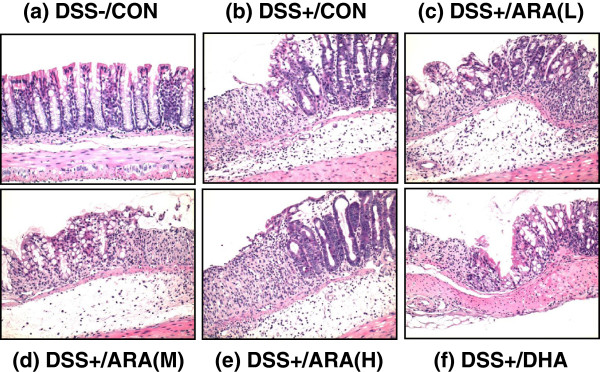
**Histology of colonic tissue in mice with DSS-induced colitis.** Colon sections were stained with H & E and representative micrographs are shown. **(a)** DSS-untreated control; DSS treatment fed with **(b)** CON diet; **(c)** ARA(L) diet; **(d)** ARA(M) diet; **(e)** ARA(H) diet; **(f)** DHA diet.

Histological analyses of colitis are shown in Figure 4. Treatment with DSS induced pathological features of mild to moderate colitis, such as crypt loss, ulceration of the mucosa, edema, and granulocyte and mononuclear cell infiltration in the mucosa. There were no significant differences in total colitis scores between the control group and the ARA- or DHA-administered group.

### Lipid mediator and gene expression

**Figure 5:**
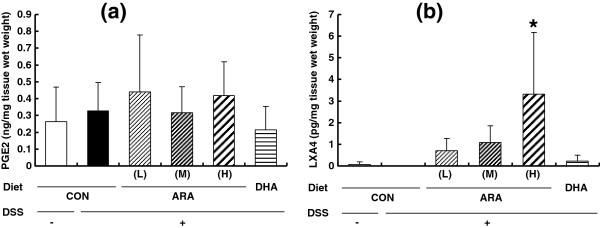
**Prostaglandin E**_**2 **_**(PGE**_**2**_**) and lipoxin A4 (LXA**_**4**_**) levels in colonic tissue of mice with DSS-induced colitis fed a CON, ARA(L), ARA(M), ARA(H) and DHA diet. a**; PGE_2_, **b**; LXA_4_. Data are represented as means ± SD. *p < 0.05 versus DSS-treated colitis group (n = 10).

We quantified PGE_2_ and LXA_4_ in the colon because they are the typical pro-inflammatory and anti-inflammatory ARA-derived lipid mediators, respectively. Treatment with DSS did not affect PGE_2_ and LXA_4_ contents in the colon (Figure 5a and b). The contents of PGE_2_ were approximately 0.2-0.4 pg/mg wet weight, and were unchanged by either of ARA or DHA administration. However, dietary ARA increased LXA_4_ content. LXA_4_ content in the ARA(H) group was 3.32 ± 2.96 pg/mg wet weight, and was significantly higher than that in the CON group (below the limit of determination). DHA administration did not affect LXA_4_ content. The contents of LTB_4_ in the colon were not different between the groups (the DSS-untreated CON group, 1.24 ± 0.39 pg/mg wet weight; the CON group, 0.96 ± 0.32; the ARA(L) group, 1.42 ± 1.20; the ARA(M) group, 1.05 ± 0.50; the ARA(H) group, 1.18 ± 0.67; and the DHA group, 0.35 ± 0.32).

We assayed the expression of genes related to lipid mediator formation, such as cPLA2, COX-1, COX-2, 5-LOX and 12/15-LOX (Additional file 4: Figure S3 a-e). Gene expression of cPLA2 and COX-2 was significantly lower after colitis induction. Gene expression of cPLA2, COX-1 and COX-2 was unchanged, but that of 5-LOX and 12/15-LOX was tend to and significantly higher after ARA or DHA administration.

### Correlation analysis

**Figure 6:**
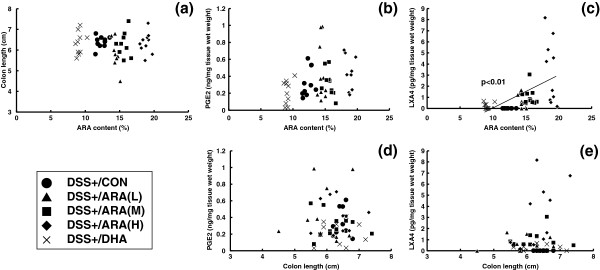
**Correlation analysis.** Correlation analyses were performed between colonic ARA contents versus colon length (disease marker) **(a)**, PGE_2_ contents **(b)**, LXA_4_ contents **(c)** and between colon length versus PGE_2_ contents **(d)**, LXA_4_ contents **(e)**.

In order to elucidate the relationship among ARA composition, contents of ARA-derived lipid mediators and colitis severity, we analyzed the correlation of these parameters in the DSS-treated group. Among the correlations between ARA composition and colon length (Figure 6a), between ARA composition and contents of ARA-derived lipid mediators (PGE_2_ (Figure 6b) and LXA_4_ (Figure 6c)) and between contents of ARA-derived lipid mediators and colon length (PGE_2_ (Figure 6d) and LXA_4_ (Figure 6e)), only ARA composition and LXA_4_ contents showed a significant positive correlation (p < 0.01).

## Discussion

In the present study, dietary ARA supplementation significantly increased ARA content in the colon in a dose-dependent manner, but did not affect colonic inflammation in a DSS-induced murine colitis model. These results suggest that ARA intake does not affect colitis. In the previous studies, there have been reported that ARA contents increased colitis and bowel disease [20,21] and they suggested that ARA exacerbates colitis. On the other hands, Ramakers et al. [14] reported that an ARA-enriched diet increased colonic ARA content in a DSS-induced murine colitis model, but did not result in more colonic inflammation as compared with fish oil- and oleic acid (OA) -enriched diets. It is interesting that the same group reported that ARA but not EPA and OA activates NF-kappaB and elevates ICAM-1 expression in Caco-2 cells [22]. Therefore, we carefully adjusted the experimental conditions of the colitis model, in which both suppression and exacerbation of colitis symptoms can be detected (Additional file 5: Supplemental Table S2).

Ramakers et al. [14] noted that intake of ARA ethyl ester improved body weight loss and diarrhea in the DSS-induced mouse colitis model. The experimental conditions of colitis induction and the severity of the colitis appear to be very similar. The main differences between the two studies were the lipids in the diets, and the chemical form and dose of ARA. With regard to lipids in diets, we adjusted the amounts of total lipids, total saturated fatty acids (S), total monounsaturated fatty acids (M), total polyunsaturated fatty acids (P), total n-6 fatty acids, and total n-3 fatty acids to similar among the groups. Therefore, the ratios of S:M:P and n-6:n-3 were automatically adjusted (approximately 1:1:1 and 2:1, respectively) in all the diets. On the other hand, the amounts of S, M, P, n-6 and n-3, and the ratios of S:M:P and n-6:n-3 were different among the groups in the previous study. This difference in diet may have resulted in the differences in the results of the two studies, as the ratios of S:M:P and n-6:n-3 affect systemic status.

The second possibility is the chemical form and dose of ARA. These differ in the two studies (present study: ARA-enriched triacylglycerol and 0.305% ARA equivalent in the diet (ARA(H) group); previous study: ARA ethyl ester and 0.78% ARA equivalent in the diet), and may have affected the increase in ARA composition in colon (present study: 12% (control group) and 18% (ARA(H) group); previous study: 15.8% (control (OA)) and 23.6% (AA group)). This might have led to the difference in the results of the two studies. However, the increases in ARA composition in the colon are sufficiently large in both studies. The present study also confirms an ARA dose-dependent increase in ARA composition in the colon in colitis. Taken together, the results of these studies suggest that dietary ARA does not exacerbate colitis in this model, and that the protective effects of dietary ARA on the colitis model are relatively small, if they exist.

This is the first study to demonstrate the relationship between ARA composition and ARA-derived lipid mediators in a colitis model. Several ARA-derived lipid mediators are known to be related to colitis. PGE_2_ has a protective role as a proliferative factor in the recovery of damaged colonic epithelial tissues. The reason why NSAID administration is avoided in colitis patients is that NSAIDs inhibit PGE_2_ production and mucosal maintenance [5-7,23]. It is also reported that administration of PGE_2_ or its analogues can alleviate colitis symptoms [24-27]. On the other hand, PGE_2_ is known to be proinflammatory for various types of acute inflammation, and may accelerate the inflammatory process by activating macrophages or fibroblasts [1]. Another ARA-derived lipid mediator, LXA_4_, is produced by 15- and 5-lipoxygenases and is clarified to have an anti-inflammatory role [2]. In the colitis model, it was reported that LXA_4_ analogue administration can protect this model [28]. Therefore, it is uncertain whether the increase in ARA content in the colon influences the contents of ARA-derived lipid mediators in the colon or colitis severity. Surprisingly, the present study revealed that the increase in ARA composition did not influence PGE_2_ content in the colon under the conditions used in the colitis model. On the other hand, LXA_4_ content in the colon increased with ARA dose. The increase in LXA_4_ contents was approximately 3.3 pg/mg colon weight in the ARA(H) group. Based on the observation that the severity of colonic inflammation was unchanged in the ARA(H) group, the increase in LXA_4_ contents observed in the present study may be not sufficient to show a marked anti-inflammatory effect in this model. Formation of higher concentrations of LXA_4_ could result in anti-inflammatory effects, as high doses of LXA_4_ analogues in drinking water (10 μg/ml 15-epi-16-parafluoro-LXA_4_) were able to reduce the severity of DSS-induced colitis [28].

It should be recognized that we quantified ARA-derived lipid mediators only at the final time point. DSS treatment continued for 7 days, whereas various physiological or pathological responses to the DSS treatment began at the first or second day. Further studies are necessary to evaluate the responses of lipid mediators at earlier stages, and to clarify the roles of lipid mediators in colitis development and recovery.

We clarified that the amounts of two ARA-derived lipid mediators, PGE_2_ and LXA_4_, show the different responses to increases in the ARA composition of colon phospholipids. This indicates that the amounts of ARA-derived lipid mediators are not determined simply by ARA composition in the colon. The reasons are unclear, but may be due to separate control of the synthetic pathways for PGE_2_ and LXA_4_. In fact, gene expression of COX-1 and COX-2, which is related to PGE_2_ synthesis, was unchanged by dietary ARA, whereas that of 12/15-LOX and 5-LOX, which are related to LXA_4_ synthesis, was increased by dietary ARA. The changes in gene expression are consistent with the changes of PGE_2_ and LXA_4_ contents. However, it is uncertain whether the changes in the expression of these genes actually contribute to the changes in PGE_2_ and LXA_4_ contents. It should also be noted that we quantified ARA-derived lipid mediators only at the final time point, similarly to the quantification of lipid mediators. Time courses of their expression are expected to be clarified in a future study.

DHA, EPA or fish oil was reported to be effective against colitis in both animal and clinical studies [29-33], while other studies failed to show any benefit [34-36]. In the present study, fish oil administration significantly increased the compositions of DHA and EPA in the colon, but did not affect inflammatory parameters other than hemoccult score at day 6 and spleen weight. The present data are consistent with the previous study; DHA composition in the colon of the DHA and CON groups was 9.8% and 6.7%, respectively, in the present study, and that of the fish oil and control groups was 9.8% and 7.1%, respectively, in the previous study [14]. Colitis symptoms were not clearly improved in either study. The DHA increase in the colon may be insufficient for improving colitis. Also, adjustment of the ratios of S:M:P and n-6:n-3 may be another reason that fish oil showed no effect. We shifted the balance of fatty acid composition in the colon by administration of ARA or DHA, but colitis severity was unchanged. This suggests that dietary PUFAs may not be a critical factor in the DSS-induced colitis model. Concerning PGE_2_ and LXA_4_ levels, either level was not affected significantly by DHA administration, but tended to be low in the DHA group. The tendency of low PGE_2_ and LXA_4_ levels is reasonable because n-3 PUFAs compete with ARA metabolisms. Further studies were needed to clarify the effect of DHA on PGE_2_ and LXA_4_ levels in colitis models.

Leukotrienes are also known as proinflammatory mediators. Synthesis of LTB_4_, one of the major leukotrienes, was shown to be enhanced in the colon of the patients with inflammatory bowel disease [30,37,38]. In the present study, LTB_4_ contents in the colon were not different between the groups, and ARA or DHA administration did not affect LTB_4_ contents. These results suggest that fatty acid composition of the colon has little impact on LTB_4_ contents in the colon under the conditions. However, LTB_4_ contents in the DHA group appear to be low, and further studies are expected to reveal the relation of DHA administration and LTB_4_ contents in colitis.

Dietary ARA intakes in the ARA(L), ARA(M) and ARA(H) groups were estimated to be approximately 75, 150 and 305 mg/kg/day, respectively, on the assumption that mice consumed about 10% of their body weight daily. In humans, the average of ARA intake from daily foods is approximately 150–200 mg ARA per day [39]. As compared to the ARA intake of humans, the ARA doses used in the present study are markedly higher. The results in the present study may thus be regarded as those under excess administration.

## Conclusion

Dietary ARA supplementation significantly increased the contents of ARA and LXA_4_ in the colon in an ARA dose-dependent manner, but did not affect colonic inflammatory parameters or PGE_2_ content in a DSS-induced murine colitis model.

## Competing interests

NT, SK, HK and HS are employees of Suntory Wellness Ltd., which is a manufacturer of foods including ARA-enriched edible oil. IM has consultancy relationships with Suntory Wellness Ltd.

## Authors’ contributions

NT, HK, HS and IM designed the study. NT and SK performed experiments and analyzed the data. NT, SK and HK drafted the manuscript, and HS and IM reviewed the manuscript. All authors read and approved the manuscript.

## Supplementary Material

Additional file 1: Figure S1 Body weight change before colitis induction. Mice consumed each diet for 42 days before DSS colitis induction. Data are represented as means ± SD (n = 10). Significant difference (*p < 0.05) versus DSS-treated colitis group was observed at 7, 17, 24, 28, 35 and 38 days (DSS+/ARA(M) group), at 35 days (DSS+/ARA(H) group) and at 17 and 31 days (DSS+/DHA group). Significant difference (**p < 0.01) versus DSS-treated colitis group was observed at 38 and 42 days (DSS+/ARA(H) group) and at 7, 14, 35, 38 and 42 days (DSS+/DHA group). Click here for file

Additional file 2: Figure S2 Body weight change ratio after colitis induction. Colitis was induced by 2% (w/v) DSS in drinking water for 7 d. Body weights were measured and expressed the percentage before DSS colitis induction. Data are represented as means ± SD. Significant difference (*p < 0.05) versus DSS-treated colitis group was observed at 3 day (DSS+/ARA(M) group) and at 6 day (DSS-/CON group). Significant difference (**p < 0.01) versus DSS-treated colitis group was observed at 3 and 7 days (DSS-/CON group). Click here for file

Additional file 3: Table S1 Effects of CON, ARA(L), ARA(M), ARA(H) and DHA diet on symptomatic marker in the murine DSS-induced colitis. Click here for file

Additional file 4: Figure S3 Expression of genes related to lipid mediator formation. cPLA2(a), COX-1(b), COX-2(c), 5-LOX(d) and 15-LOX(e) in colonic tissue of mice without DSS or with DSS-induced colitis fed CON, ARA(H) and DHA diet. Data are represented as the means ± SD. *p < 0.05 versus DSS-treated colitis group (n = 8–10). Click here for file

Additional file 5: Table S2 Preliminary investigation of DSS-induced murine colitis. Click here for file
