# Supplementary figures and images for: Dietary supplementation of arachidonic acid increases arachidonic acid and lipoxin A4 contents in colon, but does not affect severity or prostaglandin E2 content in murine colitis model
Source: Lipids Health Dis. 2014 Feb 10;13:30. doi: 10.1186/1476-511X-13-30 (PMC3928921; doi:10.1186/1476-511X-13-30)

## Slide 1
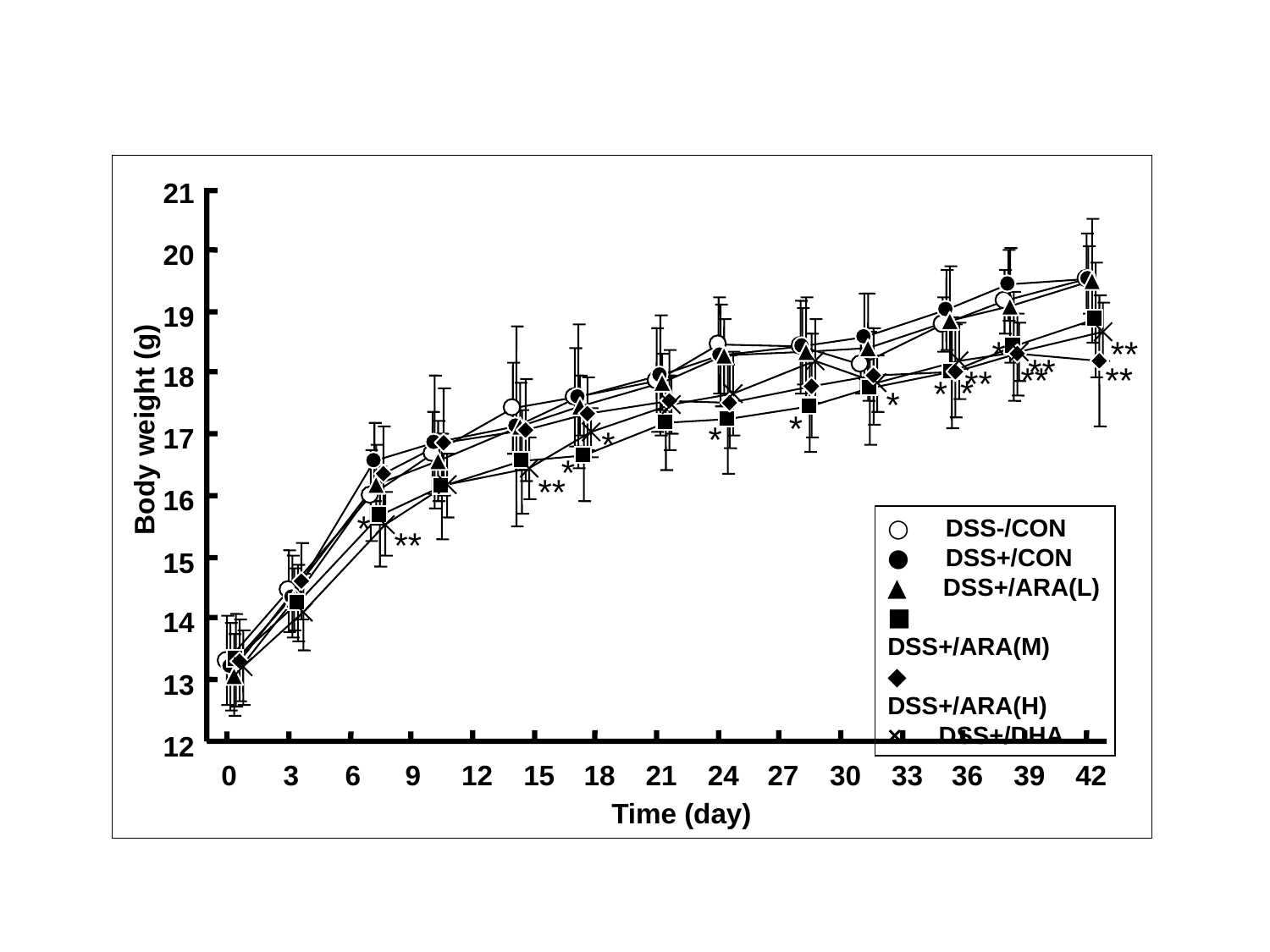

21
20
19
*
**
**
**
**
**
18
*
*
*
*
*
Body weight (g)
*
17
*
**
16
*
○　DSS-/CON
●　DSS+/CON
▲　DSS+/ARA(L)
■　DSS+/ARA(M)
◆　DSS+/ARA(H)
×　DSS+/DHA
**
15
14
13
12
0
3
6
9
12
15
18
21
24
27
30
33
36
39
42
Time (day)

Supplement: Additional file 1: Figure S1 — Body weight change before colitis induction. Mice consumed each diet for 42 days before DSS colitis induction. Data are represented as means ± SD (n = 10). Significant difference (*p < 0.05) versus DSS-treated colitis group was observed at 7, 17, 24, 28, 35 and 38 days (DSS+/ARA(M) group), at 35 days (DSS+/ARA(H) group) and at 17 and 31 days (DSS+/DHA group). Significant difference (**p < 0.01) versus DSS-treated colitis group was observed at 38 and 42 days (DSS+/ARA(H) group) and at 7, 14, 35, 38 and 42 days (DSS+/DHA group). [file 1476-511X-13-30-S1.ppt]

## Slide 1
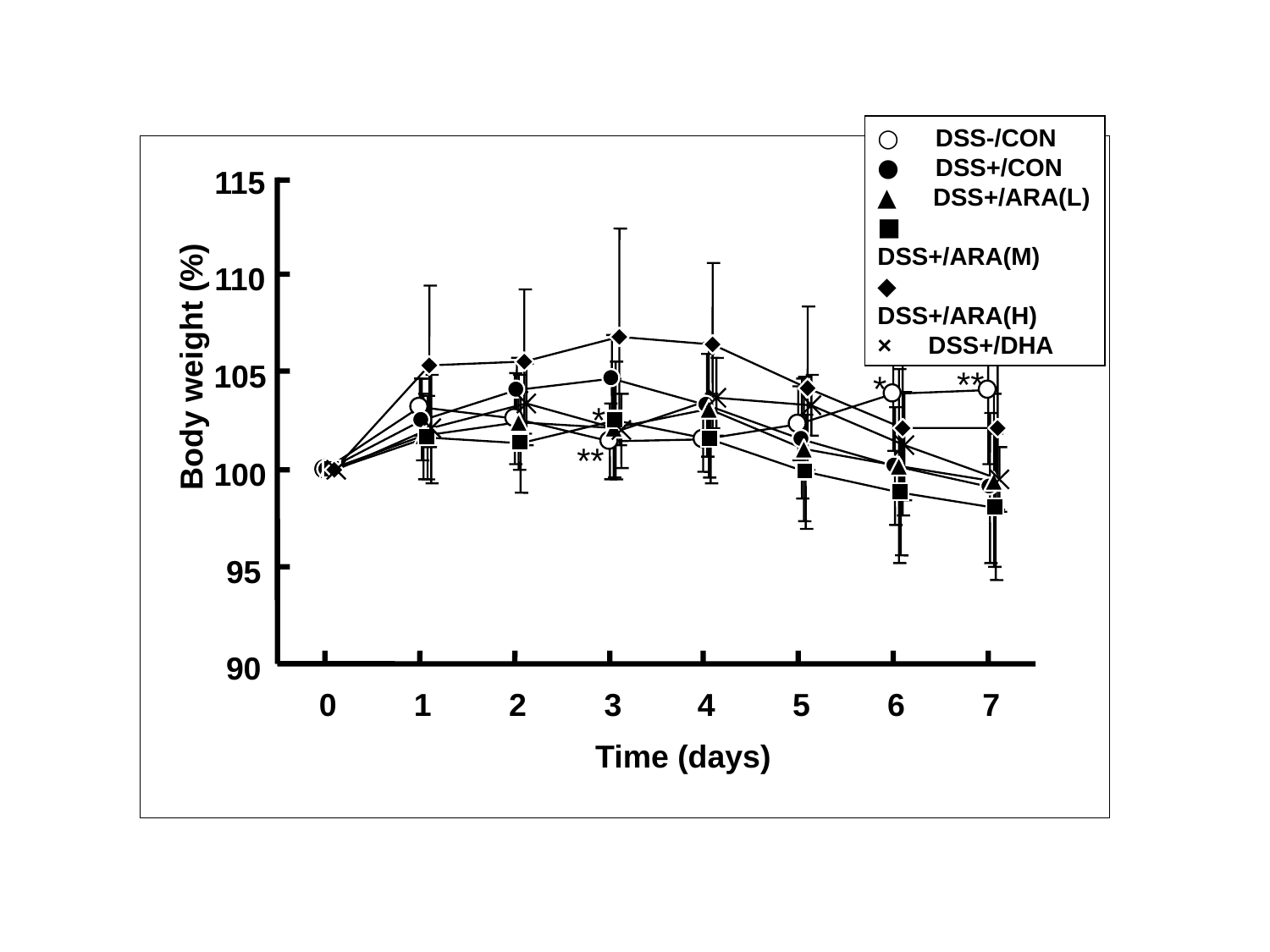

○　DSS-/CON
●　DSS+/CON
▲　DSS+/ARA(L)
■　DSS+/ARA(M)
◆　DSS+/ARA(H)
×　DSS+/DHA
115
110
Body weight (%)
**
105
*
*
**
100
95
90
0
1
2
3
4
5
6
7
Time (days)

Supplement: Additional file 2: Figure S2 — Body weight change ratio after colitis induction. Colitis was induced by 2% (w/v) DSS in drinking water for 7 d. Body weights were measured and expressed the percentage before DSS colitis induction. Data are represented as means ± SD. Significant difference (*p < 0.05) versus DSS-treated colitis group was observed at 3 day (DSS+/ARA(M) group) and at 6 day (DSS-/CON group). Significant difference (**p < 0.01) versus DSS-treated colitis group was observed at 3 and 7 days (DSS-/CON group). [file 1476-511X-13-30-S2.ppt]

## Slide 1
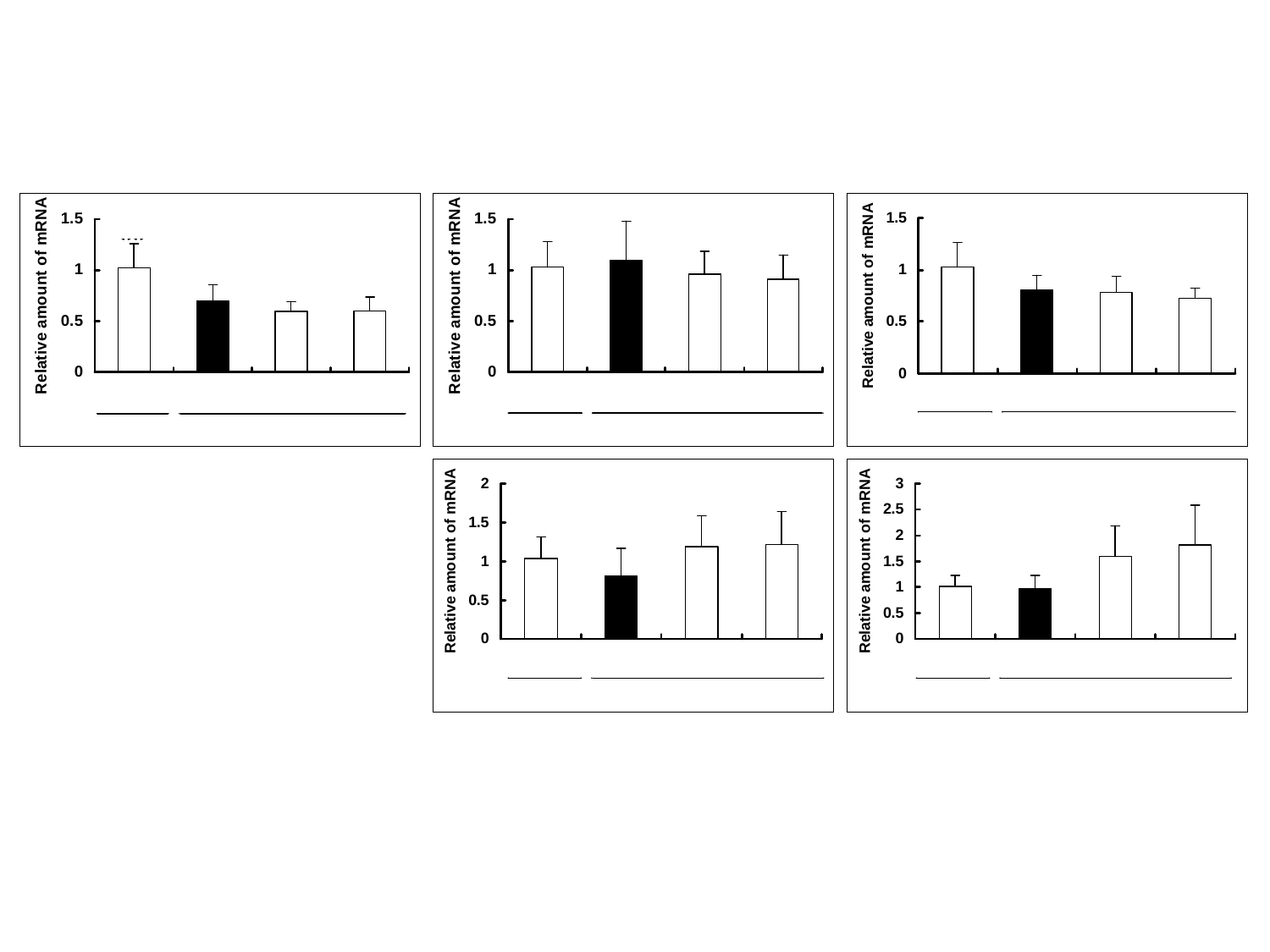

Supplement: Additional file 4: Figure S3 — Expression of genes related to lipid mediator formation. cPLA2(a), COX-1(b), COX-2(c), 5-LOX(d) and 15-LOX(e) in colonic tissue of mice without DSS or with DSS-induced colitis fed CON, ARA(H) and DHA diet. Data are represented as the means ± SD. *p < 0.05 versus DSS-treated colitis group (n = 8–10). [file 1476-511X-13-30-S4.ppt]
